# 

**Published:** 1888-12-08

**Authors:** 


### CONTENTS.

**Something about Leprosy** - - - - - 145

**Hospitals Amongst the Ancient Greeks** - - - 146

**The Indian National Association for Supplying Female Medical Aid to the Women of India, and the Countess Dufferin's Fund.** By Sir W. MOORE, K.C.I.E. - - - - - - - - - - 147

**The Dundee Infirmary.**—By ANGUS FRASER, M.D. - 148

**The Doctor's Pulpit.**—Present-Day Problems - - - 149

**Within the Wards.**—Hand-Fed Children, etc. - - 150

**Voyages in Vacation.**—XI. Russian Palaces - - - 151

**Notes and News** - - - - - - - 152

**Everybody's Page** - - - - - - - 154

**Annotations.**—The Slaughter of Shop-assistants.—The State and the Shop.—Washing Bills: A Suggestion - - - 155

**Sir Sydney Waterlow's Paper.**—An Examination of Some of its Contents - - - - - - - - 156

**False Impressions.** By CAROLINE FOTHERGILL,—Chap. X. 157

**Turning over New Leaves.**—"Heroes of Every Day Life," [illegible] "The Law of Liberty" - - - - - - 159

**Scraps and Gleanings—A Case for Help** - - 160

**EXTRA SUPPLEMENT.—THE NURSING MIRROR.**

**En Passant.**—Another Absent Nurse.—Mr. "Punch" on a Nurse's Position.—Nursing Lessons in Schools.—Our Daily Bread.—Short Items.—A Welcome Conversazione, etc. - xxxviii

**Notes from Australia** - - - - - - xxxviii

**The Women's Hospital at Redruth** - - - xxxviii

**Wages and Washing.**—II. - - - - - - xxxix

**Everybody's Opinion.**—Trained or Not?—Menial Work.—Girl Nurses —Nurses and Presents - - - - - xxxix

**Presentations—Lectures—Appointments—Vacancies Notes and Queries** - - - - - - - xl

